# Patterns of Recurrent Thrombosis in Primary Antiphospholipid Syndrome—Multicenter, Real-Life Long-Term Follow-Up

**DOI:** 10.3389/fimmu.2022.843718

**Published:** 2022-04-19

**Authors:** Stanley Niznik, Micha J. Rapoport, Orly Avnery, Aharon Lubetsky, Soad Haj Yahia, Martin H. Ellis, Nancy Agmon-Levin

**Affiliations:** ^1^ Clinical Immunology, Angioedema and Allergy Unit, The Zabludowicz Center for Autoimmune Diseases, Sheba Medical Center, Ramat Gan, Israel; ^2^ Department of Internal Medicine “C”, Shamir Medical Center, Zerifin, Israel; ^3^ Sackler School of Medicine, Tel Aviv University, Tel Aviv, Israel; ^4^ Hematology Institute and Blood Bank, Meir Medical Center, Kfar Saba, Israel; ^5^ The National Hemophilia Center and Thrombosis Unit, Amalia Biron Research Institute of Thrombosis and Hemostasis, Sheba Medical Center, Ramat Gan, Israel

**Keywords:** antiphospholipid syndrome, thrombosis - immunology, recurrence, cardiovascular disease, AGAPSS, therapy

## Abstract

**Background:**

Antiphospholipid syndrome (APS) is an acquired hypercoagulable condition associated with antiphospholipid antibody (aPL) presence. Data on re-thrombosis following APS-diagnosis are limited.

**Methods:**

This is a retrospective analysis of new thrombotic events among primary APS (pAPS) patients followed for up to 15 years in three medical centers in Israel.

**Results:**

Among 312 primary-APS patients, 143 (46%) had new thrombotic event classified to three patterns: (1) *Arterial—*associated with heart valve disease (OR 7.24, 95% C.I. 2.26–24.6), hypertension (OR 3, 95% C.I. 1.44–6.25), elevated anti-B2-GPI IgM (OR 1.04, 95% C.I. 0.996–1.08), arterial thrombosis at presentation (OR 1.74 95% C.I. 0.992–3.26), and older age (41 vs. 34 years, p < 0.001). (2) *Venous—*linked with venous thrombosis at presentation (OR 12.9, 95% C.I. 5.27–31.6, p < 0.001), heart valve disease (OR 9.81 95% C.I. 1.82–52.9, p = 0.018), aGAPSS (OR 1.15 95% C.I. 1.02–1.29), and younger age (31 vs. 36.5 years, p = 0.001); and (3) *Combined pattern*—associated with heart valve disease (OR 40.5 95% C.I. 7.7–212) and pulmonary embolism (OR 7.47 95% C.I. 1.96–28.5). A 4th variant “the Breakthrough pattern” defined by re-thrombosis despite prophylactic therapy was observed in 100/143 (70%) patients and linked with heart valve disease (OR 8. 95% C.I. 2.43–26.3), venous thrombosis at presentation (OR 2.61 95% C.I. 1.47–4.66), leg ulcers (OR 12.2, 95% C.I. 1.4–107), hypertension (OR 1.99, 95% C.I. 0.92–4.34), and higher aGAPSS (OR 1.08, 95% C.I. 0.99–1.18).

**Conclusion:**

In this real-life observation, re-thrombosis was common among pAPS patients including in those recommended to receive prophylactic therapy. Different patterns of recurrence were identified and linked with presenting symptoms, specific serological markers, APS manifestations, and comorbidities. Studies that will address interventions to prevent recurrences of APS-related events are needed.

## Introduction

The antiphospholipid syndrome (APS) is an autoimmune acquired coagulopathy characterized by thrombosis and obstetric morbidity in the presence of antiphospholipid antibodies (aPLs). Since first described in the 1980s, classification criteria of APS are based on the concomitant presence of typical clinical features and aPLs. These criteria, though designed for classification, are often used for diagnosis (1). Additional features currently defined as the “non-criteria” manifestations, such as thrombocytopenia, autoimmune hemolytic anemia (AIHA), heart valve disease, and non-thrombotic neurological manifestations, have been related to APS (2). Lastly, an aggressive subset of this disease termed catastrophic APS (cAPS) is documented in 1% of patients and withhold poor outcomes (2, 3).

Factors predictive of APS course and prognosis are not well defined, and upon APS diagnosis treatment protocols typically follow general guidelines comprising antiplatelet and/or anticoagulants (4). Recurrent APS-related events are common and difficult to predict (5–9). Thereby, the Global Antiphospholipid Score (GAPSS) was developed for the assessment of thrombosis risk in APS. This score include 6 factors, namely, seropositivity for anti-cardiolipin, anti-B2GPI, lupus anticoagulant, and anti-phosphatidylserine–prothrombin complex antibodies, as well as dyslipidemia and hypertension (10). The GAPSS score is rarely used because of the scarcity of laboratories performing the anti-phosphatidylserine–prothrombin complex assay and is commonly replace by a modified GAPSS (aGAPSS) (11). The latter has been validated in several cohorts; nonetheless, it has some limitations such as the simplicity of aPL assessment with no isotype or titer measurement as well as the lack of non-criteria manifestations. Other risk factors have related to recurrence of APS manifestation such as the presence of LAC or triple aPL positivity (i.e., the concomitant presence of anti-cardiolipin (aCL) and anti-β_2_‐glycoprotein I (aβ_2_GPI) and lupus anticoagulants (LAC) (8, 12). Of note, the occurrence of arterial thrombosis has long been considered a risk factor of aggressive disease, with some advocating a more intensive treatment, although this is not a matter of consensus (4, 13–15).

Currently, the risk of APS recurrence is difficult to assess in general and particularly at presentation of disease. In this multicenter study, we evaluated the associations between features presented early/at diagnosis of primary APS, and recurrence of thrombotic events during the long-term follow-up.

## Patients and Methods

### Patients

This is a retrospective study of primary APS patients diagnosed according to the international (Sydney) classification criteria for the antiphospholipid syndrome (1). Data were retrieved from medical records of sequential patients treated in three large centers in Israel (Sheba-Tel Hashomer, Meir, and Shamir Medical Centers) during January 2004 to December 2019. This study was performed in accordance with the declaration of Helsinki and in agreement and approved for this study by the Sheba Medical Center Review Board.

Patients who at presentation of APS or at any point of the disease fulfilled the criteria of systemic lupus erythematous disease, based on the relevant diagnostic criteria (16, 17), or another systemic autoimmune disease were excluded. All patients were treated in specialized centers, and decisions upon follow-up and therapy were at their specialist discretion.

Temporal associations were established between the first clinical event which led to APS diagnosis (i.e., presenting symptom) and recurrent thrombosis. For this purpose, three clinical patterns of recurrence during follow-up were defined: [1] “arterial pattern” (e.g., stroke, limb ischemia, myocardial infarction); [2] “venous pattern” (i.e., deep vein thrombosis, pulmonary embolism presented); [3] “combined pattern” (APS-related events of mixed origin during follow-up, i.e., arterial and obstetric; venous and obstetric; arterial and venous; and arterial, venous, and obstetric). Additionally, a fourth variant, the “breakthrough pattern”, was defined by recurrence of thrombotic events despite recommended anti-thrombotic therapy, regardless of type of thrombosis. Patients with each recurrence pattern were compared with primary APS patients who have had no thrombosis during follow-up.

Demographic characteristics (age, sex, age at diagnosis, length of follow-up, treatments); presenting APS classification clinical criteria (i.e., thrombotic or obstetric events and aPL serology); concomitant conditions (hypertension, smoking, diabetes mellitus, and dyslipidemia); non-criteria APS-related manifestations manifesting at any time during the disease course (heart valve disease (Libman–Sacks endocarditis), livedo reticularis, leg ulcers, migraine, epilepsy, autoimmune hemolytic anemia, thrombocytopenia, leukopenia); APS-related outcomes (death, catastrophic APS, aGAPSS, bleeding events); and therapies prescribed at any point of the disease were also collected.

### Serology and Scores

The presence of anti-cardiolipin (aCL) and anti-β_2_‐glycoprotein I (aβ_2_GPI) of the IgG and IgM isotypes was measured by enzyme‐linked immunosorbent assay (ELISA) or by a multiplex system. The kits that were used were all commercial (ELISA—aB2GPI by AESKU Diagnostics (Wendelsheim, Germany) and aCL by Varelisa (Pharmacia Diagnostics, Stockholm, Sweden); Bioplex both aB2GPI and aCL by Bio-Rad, Hercules, CA, USA). B2GPI and ACL were considered positive if antibody levels were above 20 MPL units (IgG phospholipid units or IgM phospholipid units), or if >99th percentile or according to the manufacturer’s instructions were present in a minimum of two tests performed at least 12 weeks apart were obtained. Very high titers were considered as fourfold or higher of the upper normal limit as specified for each kit. Lupus anticoagulant (LA) activity was detected by coagulation assays in routine use at each center and was consistent with the International Society of Thrombosis and Hemostasis guidelines (18). The LA assays were modified in 2016; up until 2016, LA activity was measured by LA-responsive activated partial thromboplastin time (aPTT) aPL (by Stago, confirmed using the Actem FS Kit by Siemens, Erlangen, Germany), and from 2016, LA activity was measured by a combination of silicon clotting time and the use of the Russell Viper Venom Kit (by IL, Bedford, MA, USA). In case of anticoagulation treatment or spontaneous INR >1.5, patients’ plasma was mixed with normal plasma in order to reduce false positivity. Positivity was defined as single, double, or triple positive according to the number of different positive tests obtained.

In this study, we used the validated aGAPSS (11, 19) which allots 3 points for dyslipidemia, 1 for arterial hypertension, 5 for anti-cardiolipin antibodies IgG/IgM, 4 for anti-β2 glycoprotein IgG/IgM, and 4 for lupus anticoagulant. Catastrophic APS (cAPS) was defined according to the International Task Force on CAPS criteria (2).

### Statistical Analysis

The data were analyzed using BMDP software (BMDP Statistical Software, University of California Press, Berkeley, LA, USA). Pearson’s chi-square test or Fisher’s exact test (two-tailed) was used for the analysis of between-group differences in discrete variables, and analysis of variance (ANOVA) was used for comparing continuous variables. Using those variables found to be significant (<0.10) on univariate analysis, we applied a stepwise logistic regression in order to determine those variables most significantly associated with each outcome. Choices of variables to include in our stepwise analysis were drawn from the number of patients who were to prognosticate; thus, for every 5 patients, 1 variable was allowed. Patients with missing relevant data were excluded from the analysis. Odds ratios (ORs) with 95% confidence intervals (CIs) were calculated, and a p-value of ≤0.05 was considered significant.

## Results

We included 312 primary APS patients in this study. The combined duration of follow-up was 3,182 patient-years (mean 10 ± 7 years; **Table 1**). APS-related events during follow-up (excluding the initial “classification” event) were documented in 180 (57.6%) patients, of whom 143/180 (79%) experienced an additional thrombotic event while in 37/180 (21%) female patients’ obstetric complications were the recurrent event. Thus, 169/312 (54.2%) patients that had no thrombotic event during follow-up were compared to different patterns of thrombotic recurrences. There were 100 among 143 (70%) patients who suffered from recurrent thrombosis despite preventive therapy. Notably in our entire cohort, 95.5% (298/312) received guideline-based therapy (**Table 1**). During the entire study period, 31 bleeding events were reported in 27/312 (8.6%) patients, of which 16/312 (5.1%) were defined as major bleeding resulting in a bleeding rate of 0.97 events/100 patient years.

**Table 1:** General features of patients with primary antiphospholipid syndrome.

**Parameter**	**Primary APS (N = 312)**
Age at presentation ( ± SD)	36 ( ± 13.5)
Male gender %	93 (29.8%)
Years of follow-up ( ± SD)	10 ( ± 7)
**Presenting symptom**	
Arterial thrombosis	122 (39.1%)
Venous thrombosis	120 (38.4%)
Obstetric morbidity	70 (22.4%)
**Serology**	
Single positivity	50 (16.2%)
Double positivity	87 (27.8%)
Triple positivity	175 (56.1%)
**Thrombotic event recurrence*^a^***	
Arterial events	76 (24.3%)
Venous events	48 (15.3%)
Combined events*^b^*	19 (6.1%)
Any events despite preventive therapy*^c^*	100 (32.1%)
**APS-related outcomes**	
aGAPSS	11.1 ( ± 3.6)
Death	9 (2.8%)
cAPS	7 (2.2%)
Overall bleeding	27 (8.6%)
Major bleeding	16 (5.1%)
Minor bleeding	11 (3.5%)
**Therapy**	
* No therapy*	14 (4.5%)
* Intermittent! LMWH + antiplatelet*	49 (15.7%)
Antiplatelet (only)	47 (15%)
LMWH 0.5 mg/kg ×2 (only)	14 (4.5%)
LMWH 1 mg/kg ×2 (only)	20 (6.4%)
Warfarin (only)	77 (24.7%)
LMWH + antiplatelet	33 (10.6%)
Warfarin + antiplatelet	58 (18.6%)

^a^Thrombotic events that occurred after establishment of APS diagnosis.

^b^Thrombotic events of combined nature occurred in the same patients (i.e., arterial and venous, venous and obstetric, obstetric and arterial, and arterial, obstetric and venous).

^c^Clinical and serological parameters associated with new APS-related event despite preventive therapy (anti-aggregation and/or anticoagulation).

APS, antiphospholipid syndrome; SD, standard deviation; aGAPSS, adjusted Antiphospholipid Syndrome Score; cAPS, catastrophic antiphospholipid syndrome; LMWH, low molecular weight heparin; Intermittent LMWH, therapy given intermittently, e.g., peripartum, peri-surgery.

**Figure 1:**
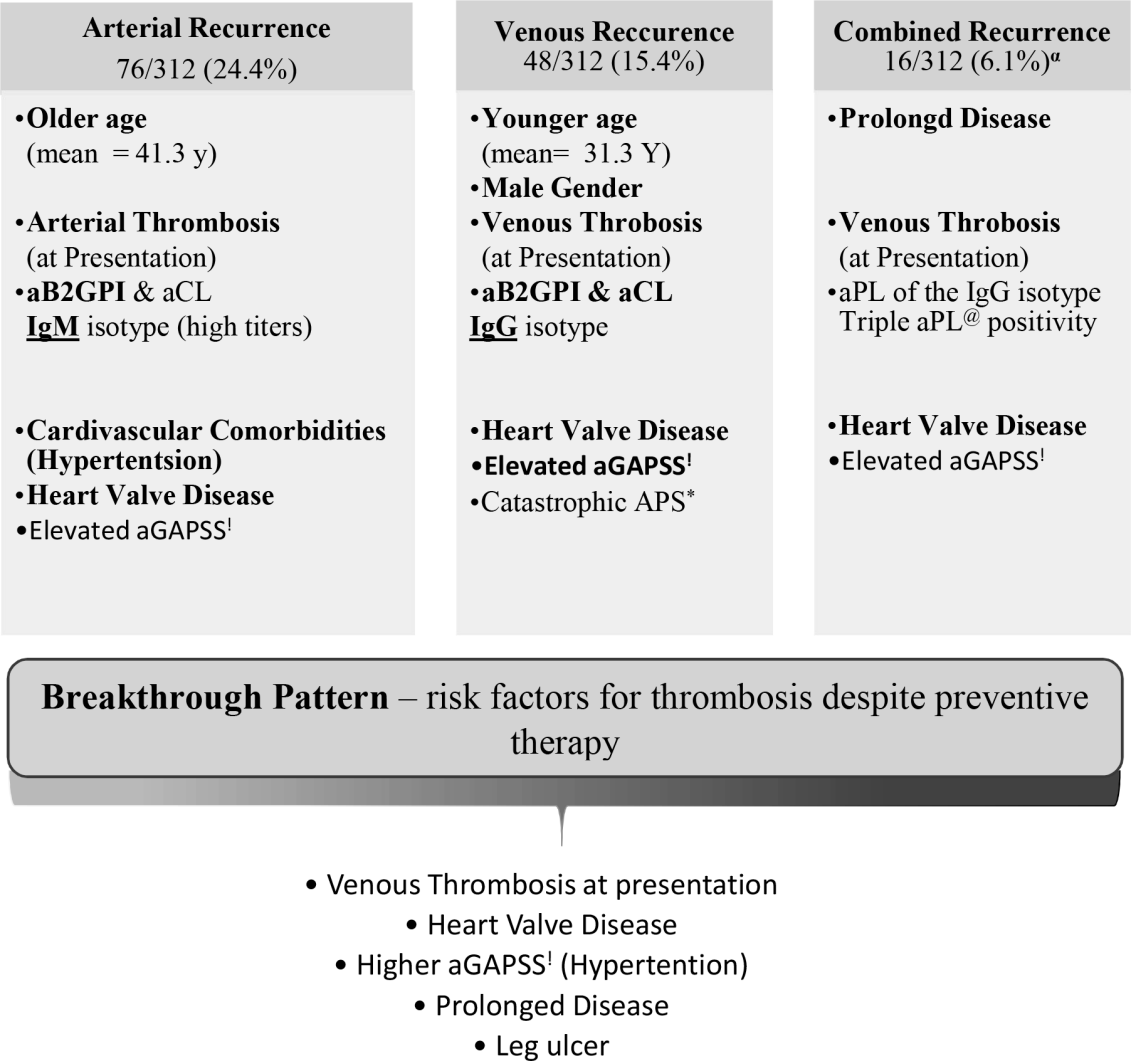
Patterns of thrombotic recurrence in the primary Antiphospholipid Syndrome (n=143). All patients with at least one thrombotic event after established antiphospholipid syndrome. ^α^Subset of patients with multiple types of thrombosis (i.e., arterial and venous; arterial and obstetric; venous and obstetric; arterial, venous and obstetric). ^*^Antiphospholipid. ^@^Antiphospholipid antibody.^!^Adjusted Global Antiphospholipid Syndrome.

Four distinct patterns of new thrombotic events during follow-up were identified in this study, namely, “arterial”, “venous”, “combined”, and “breakthrough” (**Figure 1**). Notably, longer follow-up was linked with more recurrence regardless of the pattern.

*The “arterial” pattern* was documented in 76/312 (24.4%) primary APS (pAPS) patients (**Table 2**). These events were associated with older age (41.3 ± 13.2 vs. 34.2 ± 13.2; p < 0.001) and occurred mainly among patients with arterial thrombosis at APS diagnosis. Arterial thrombosis during follow-up was also more common in patients with very high titer (>4-fold upper normal limit) aPLs of the IgM isotype (both anti-B2-GPI and anti-cardiolipin) hypertension, dyslipidemia, heart valve disease, higher aGAPSS, and higher mortality in comparison to patients with no recurrence during follow-up (**Table 2**). Interestingly, when comparing aPLs among patients with the arterial pattern of recurrence to our entire cohort of primary APS patients (including those with other patterns of re-thrombosis), the link with aPLs of the IgM isotype remained persistent regardless of titers (B2GPI IgM 64.5% vs. 43.6%, p = 0.002, aCL IgM 56.5% vs. 39% p = 0.001). In the stepwise regression analysis, the most important factors related to the “arterial” pattern of recurrence were heart valve disease (OR 7.24, 95% C.I. 2.26–24.6), hypertension (OR 3, 95% C.I. 1.44–6.25), elevated anti-B2-GPI IgM (OR 1.04, 95% C.I. 0.996–1.08), and arterial thrombosis at presentation (OR 1.74 95% C.I. 0.992–3.26), with area under the curve of 0.726.

**Table 2:** Clinical and serological parameters associated with the “arterial pattern”.

Parameter	Arterial thrombosis recurrence (N = 76)	No thrombotic recurrence (N = 169)	p value
Age at diagnosis (years ± SD)	41 ( ± 13)	35 ( ± 13)	0.001
Male	18 (24%)	45 (26.6%)	0.628
Average follow up (years ± SD)	11.5 ( ± 8)	8.7 ( ± 6.6)	<0.001
Presenting symptoms (classification criteria)			
Arterial thrombosis	47 (61.8%)	67 (39.6%)	<0.001
Venous thrombosis	3 (3.9%)	53 (31.3%)	<0.001
Obstetric manifestation	28 (21.2%)	49 (28.9%)	0.015
Serology			
Anti-B2-GPI (IgM)	46 (60.5%)	86 (49.7%)	0.16
Anti-B2 GPI (IGM) high titer (>4 times upper normal limit)	20 (26.3%)	22 (13%)	<0.001
Anti-B2-GPI (IgG)	49 (64.5%)	86 (56.8%)	0.069
Anti-B2-GPI (IgG) (>4 times upper normal limit)	28 (36.8%)	57 (33.7%)	0.248
Anti-cardiolipin (IgM)	43 (56.5%)	75 (44.3%)	0.077
Anti-cardiolipin (IgM) (4 times upper normal limit)	16 (21.1%)	19 (11.2%)	0.04
Anti-cardiolipin (IgG)	47 (61.8%)	111 (65.6%)	0.561
Anti-cardiolipin (IgG) high titer (4 times upper normal limit)	26 (35.1%)	59 (34.9%)	0.325
Lupus anticoagulant	55 (72.4%)	112 (66.2%)	0.343
Triple positivity	45 (59.2%)	78 (46.1%)	0.059
General features			
Hypertension	28 (36.8%)	20 (11.1%)	<0.001
Smoking	14 (18.5%)	22 (13%)	0.269
Diabetes mellitus	5 (6.6%)	12 (7.1%)	0.136
Dyslipidemia	19 (25%)	20 (11.1%)	0.009
Non-criteria manifestation			
Heart valve disease	13 (17.1%)	5 (2.9%)	<0.001
Livedo reticularis	4 (5.6%)	14 (8.2%)	0.375
Leg ulcer	6 (7.9%)	2 (1.1%)	0.006
Migraine	12 (15.8%)	21 (12%)	0.475
Epilepsy	5 (6.6%)	9 (5.3%)	0.696
Autoimmune hemolytic anemia	5 (6.6%)	4 (2.3%)	0.105
Thrombocytopenia	13 (17.1%)	37 (21%)	0.39
Leukopenia	4 (5.6%)	10 (5.9%)	0.838
APS-related outcomes			
CAPS	0 (0.0%)	0 (0%)	1.00
Death	5 (6.6%)	2 (1.1%)	0.019
aGAPSS	12 (± 3.6)	10.3 (± 3.7)	0.002

CAPS, catastrophic APS; aGAPSS, adjusted Global Antiphospholipid Score.

*The “venous” pattern* was observed in 48/312 (15.4%) (**Table 3**). This was associated with younger age (31.3 ± 12.7 vs. 36.7 ± 13.5 p = 0.011), male gender, and venous thrombosis at APS presentation. This pattern was also linked with anti-cardiolipin and anti-B2GPI, but of the IgG isotype, triple aPL positivity, heart valve disease, and aGAPSS. On stepwise regression analysis, the three parameters mostly related to this pattern were venous thrombosis at presentation (OR 12.9, 95% C.I. 5.27–31.6, p < 0.001), heart valve disease (OR 9.81 95% C.I. 1.82–52.9, p = 0.018), and aGAPSS (OR 1.15 95% C.I. 1.02–1.29), with area under the curve of 0.825.

**Table 3:** Clinical and serological parameters associated with the “venous pattern”.

	Venous thrombosis recurrence (n = 48)	No thrombotic recurrence (N = 169)	p value
Age at diagnosis (years ± SD)	31 ( ± 13)	35 ( ± 13)	0.06
Male	23 (48.9%)	45 (26.6%)	0.005
Average time of follow-up (years ± SD)	11 ( ± 7.5)	8.7 ( ± 6.6)	0.016
Presenting symptoms (classification criteria)			
Arterial thrombosis	4 (8.5%)	67 (39.6%)	0.013
Venous thrombosis	40 (83.3%)	53 (31.3%)	<0.001
Obstetric manifestations	6 (12.5%)	49 (28.9%)	0.014
Serology			
Anti-B2-GPI (IgM)	13 (27.5%)	84 (49.7%)	0.005
Anti-B2 GPI (IGM) high titer (4 times upper normal limit)	5 (10.6%)	22 (13%)	0.623
Anti-B2-GPI (IgG)	37 (77.1%)	86 (56.8%)	0.002
Anti-B2-GPI (IgG) (4 times upper normal limit)	26 (55.3%)	57 (33.7%)	0.016
Anti-cardiolipin (IgM)	16 (33.3%)	75 (44.3%)	0.171
Anti-cardiolipin (IgM) (4 times upper normal limit)	3 (6.8%)	19 (11.2%)	0.312
Anti-cardiolipin (IgG)	40 (83.3%)	111 (65.6%)	0.019
Anti-cardiolipin (IgG) high titer (4 times upper normal limit)	28 (59.5%)	59 (34.9%)	0.035
Lupus anticoagulant	36 (75%)	112 (66.2%)	0.252
Triple positivity	31 (64.5%)	78 (46.1%)	0.024
Cardiovascular risk factor			
Hypertension	7 (14.2%)	20 (11.1%)	0.611
Smoking	10 (21.3%)	22 (13%)	0.178
Diabetes mellitus	3 (6.4%)	12 (7.1%)	0.837
Dyslipidemia	7 (14.9%)	20 (11.1%)	0.611
Non-criteria manifestation			
Heart valve disease	5 (10.4%)	5 (2.9%)	0.023
Livedo reticularis	3 (6.4%)	14 (8.2%)	0.643
Leg ulcer	2 (4.3%)	2 (1.1%)	0.175
Migraine	4 (8.3%)	21 (12%)	0.433
Epilepsy	3 (6.4%)	9 (5.3%)	0.805
Autoimmune hemolytic anemia	5 (10.6%)	4 (2.3%)	0.013
Thrombocytopenia	10 (21.3%)	37 (21%)	0.875
Leukopenia	4 (8.3%)	10 (5.9%)	0.401
APS-related outcomes			
CAPS	5 (10.6%)	0	0.004
Death	1 (2.1%)	2 (1.1%)	0.637
aGAPSS	11.2 (± 3.3)	10.3 ( ± 3.7)	0.013

CAPS, catastrophic APS; aGAPSS, adjusted Global Antiphospholipid Score.

*The “combined” pattern* in which more than one type of APS-related event occurred during follow-up was documented in 19/312 (6.1%) patients. Combined events including arterial thrombosis and obstetric morbidity in 2 patients, arterial and venous thrombosis in 9 patients, venous thrombosis and obstetric morbidity in 4 patients, and lastly 4 patients have had all three—arterial thrombosis, venous thrombosis, and obstetric morbidity. This pattern was associated with longer disease course and follow-up by 6.3 years, venous thrombosis as the presenting symptom, and aPL triple positivity as well as IgG isotypes (**Table 4**). For this relatively more severe phenotype, we also evaluated interactions with non-criteria manifestations at any time during the course of the disease; thus notably, this pattern was associated with heart valve disease, livedo reticularis, and leg ulcers. The aGAPSS score was significantly elevated in this group (11.9 vs. 10.3 in the control group, p = 0.013). Lastly, patients with this pattern presented more often with cAPS. Using a stepwise regression analysis, we identified 2 statistically significant factors which had the most impact on this phenotype: heart valve disease (OR 40.5 95% C.I. 7.7–212) and pulmonary embolism (OR 7.47 95% C.I. 1.96–28.5), with area under the curve of 0.805.

**Table 4:** Serological Clinical parameters of OAPS patients with thrombosis (OAPSt) and without thrombosis during follow up (OAPSnt).

Parameter	OAPSt (n=24)	OAPSnt (n=43)	P Value
Anti-B2GPI ( IgM)	54.1% (13)	48.8% (21)	0.8
Anti-B2GPI (IgG)	54.1% (13)	60.5% (26)	0.8
Anti-cardiolipin (IgM)	54.1% (13)	41.9% (18)	0.33
Anti-cardiolipin (IgG)	54.1% (13)	72.1% (31)	0.2
Lupus anti-coagulant	66.6% (16)	58.1% (25)	0.5
aPL Triple positive	58.3% (14)	46.5% (20)	0.35
ANA positive	45.8% (11)	20.9% (9)	0.04
C3 (mean value) mg/dl	20.8% (5)	11.6% (4)	0.2
C4 (mean value) mg/dl	16.6% (4)	9.3% (2)	0.09

*The “breakthrough pattern”* addressed the occurrences of APS-related events despite preventive therapy in our entire cohort regardless of presenting symptoms or type of new thrombotic event. This variant was documented in 100/143 (70%) pAPS patients, of whom 81 (81%) were treated with anticoagulants ± antiplatelet agents (**Table 5**). In comparison to the patients who have had no thrombosis during follow-up, the patients with the “breakthrough pattern” were more likely to be treated with the combination of warfarin and antiplatelet agent (27% vs. 14.6%, p = 0.012) and less likely to be treated with antiplatelet therapy only (8% vs. 17.5%, p = 0.026). *The “breakthrough”* pattern was also associated with venous thrombosis at presentation, prolonged duration of disease (>4 years on average), limb ischemia/ulcers, and heart valve disease. Using a stepwise regression analysis, we identified five factors which had the strongest association with this pattern, namely, heart valve disease (OR 8 95% C.I. 2.43–26.3), venous thrombosis at presentation (OR 2.61 95% C.I. 1.47–4.66), leg ulcers (OR 12.2, 95% C.I. 1.4–107), hypertension (OR 1.99, 95% C.I. 0.92–4.34), and higher aGAPSS (OR 1.08, 95% C.I. 0.99–1.18), with area under the curve of 0.716.

**Table 5:** Clinical and serological parameters associated with the “breakthrough” pattern—e.g., APS-related event despite preventive therapy (anti-aggregation or anticoagulation).

	Recurrent thrombosis despite therapy (n = 100)	No thrombotic recurrence (N = 169)	p value
Age at diagnosis (years ± SDV)	34.6 ( ± 13)	35 ( ± 13)	0.555
Male gender %	36 (36%)	45 (26.6%)	0.577
Average follow up (years ± SDV)	12 ( ± 8)	8.7 ( ± 6.6)	<0.001
Presenting symptoms (classification criteria)			
Arterial thrombosis	38 (38%)	67 (39.6%)	0.80
Venous thrombosis	51 (51%)	53 (31.3%)	0.001
Obstetric manifestations	11 (11%)	49 (28.9%)	<0.001
Serology			
Anti-B2-GPI (IgM)	46 (46%)	84 (49.7%)	0.11
Anti-B2 GPI (IGM) high titer (4 times upper normal limit)	16 (16%)	22 (13%)	0.91
Anti-B2-GPI (IgG)	69 (69%)	86 (50.8%)	0.033
Anti-B2-GPI (IgG) (4 times upper normal limit)	51 (51%)	57 (33.7%)	0.115
Anti-cardiolipin (IgM)	30 (30%)	75 (44.3%)	0.019
Anti-cardiolipin (IgM) (4 times upper normal limit)	14 (14%)	19 (11.2%)	0.505
Anti-cardiolipin (IgG)	69 (69%)	111 (65.6%)	0.60
Anti-cardiolipin (IgG) high titer (4 times upper normal limit)	49 (49%)	59 (34.9%)	0.213
Lupus anticoagulant	76 (76%)	112 (66.2%)	0.093
Triple positivity	63 (63%)	78 (46.1%)	0.029
General features			
Hypertension	23 (23%)	20 (11.1%)	0.037
Smoking	17 (17%)	22 (13%)	0.476
Dyslipidemia	11 (11%)	12 (7.1%)	0.274
Diabetes mellitus	13 (13%)	20 (11.1%)	0.362
APS thrombotic event at follow-up			
Stroke	29 (29.0%)	37 (21.8%)	0.235
Limb ischemia	13 (13.0%)	8 (3.8%)	0.006
Deep vein thrombosis	42 (42.0%)	36 (21.3%)	<0.001
Pulmonary embolism	34 (34.0%)	18 (10.8%)	<0.001
Non-criteria manifestation at any time			
Valve disease	16 (16.0%)	5 (2.9%)	<0.001
Leg ulcer	8 (8.0%)	1 (0.6%)	0.031
Migraine	12 (12.0%)	2 (1.1%)	1.00
Epilepsy	3 (3.0%)	21 (12%)	0.28
Autoimmune hemolytic anemia	12 (12.0%)	13 (8.8%)	0.30
Thrombocytopenia	18 (18.0%)	35 (20.1%)	0.43
APS-related outcomes			
CAPS	3 (3.0%)	0 (0%)	0.06
Death	4 (4.0%)	2 (1.1%)	0.216
aGAPSS	11.6 ( ± 3.6)	10.3 ( ± 3.8)	0.0126
Therapy			
* No therapy*	0 (0%)	12 (7.1%)	<0.001
* Intermittent! LMWH + antiplatelet*	11 (11%)	41 (24.2%)	0.135
Antiplatelet (only)	8 (8%)	28 (16.5%)	0.026
LMWH 0.5 mg/kg ×2 (only)	8 (8%)	3 (1.8%)	0.074
LMWH 1 mg/kg ×2 (only)	5 (5%)	8 (4.7%)	0.623
Warfarin (only)	29 (29%)	36 (21.3%)	0.26
LMWH + antiplatelet	12 (12%)	15 (8.9%)	0.56
Warfarin + antiplatelet	27 (27%)	31 (13%)	0.012

NR, not relevant; CAPS, catastrophic APS; aGAPSS, adjusted Global Antiphospholipid Score; LMWH, low molecular weight heparin; intermittent LMWH, therapy given intermittently, e.g., peripartum, peri-surgery.

*The “breakthrough pattern”* addressed the occurrences of APS-related events despite preventive therapy in our entire cohort regardless of presenting symptoms or type of new thrombotic event. This variant was documented in 100/143 (70%) pAPS patients, of whom 81 (81%) were treated with anticoagulants ± antiplatelet agents (**Table 5**). In comparison to the patients who have had no thrombosis during follow-up, the patients with the “breakthrough pattern” were more likely to be treated with the combination of warfarin and antiplatelet agent (27% vs. 14.6%, p = 0.012) and less likely to be treated with antiplatelet therapy only (8% vs. 17.5%, p = 0.026). *The “breakthrough”* pattern was also associated with venous thrombosis at presentation, prolonged duration of disease (>4 years on average), limb ischemia/ulcers, and heart valve disease. Using a stepwise regression analysis, we identified five factors which had the strongest association with this pattern, namely, heart valve disease (OR 8 95% C.I. 2.43–26.3), venous thrombosis at presentation (OR 2.61 95% C.I. 1.47–4.66), leg ulcers (OR 12.2, 95% C.I. 1.4–107), hypertension (OR 1.99, 95% C.I. 0.92–4.34), and higher aGAPSS (OR 1.08, 95% C.I. 0.99–1.18), with area under the curve of 0.716.

## Discussion

In this study, we aimed to determine risk factors linked with recurrent thrombosis among APS patients in Israel. Focusing on primary APS (pAPS) enabled us to minimize confounding factors related to SLE or other systemic autoimmune disease activity and/or therapy. We therefore studied the course of 312 pAPS patients for an average duration of more than 10 years. During this period, 143 (46%) experienced a new thrombotic event which occurred in 100 (70%) of them despite receiving guideline-appropriate antithrombotic treatment at the time of recurrence.

Few studies have focused on thrombosis recurrence in APS, and most have included mixed populations of patients with primary and/or secondary APS. Among these studies, a thrombotic recurrence rate of 25%–30% during 4 to 10 years of follow-up has been reported (6–9, 19–21). In a recent study, with an average follow-up of 18 years, a higher rate of 44% new thrombotic events was observed (5) and stands in agreement with our prolonged study. A novel aspect of our report is the definition of patterns of recurrence, of which two patterns of recurrence termed “combined” and “breakthrough” were strongly linked with longer duration of disease. This is consistent with the Piedmont cohort in which thrombosis recurrence despite preventive therapy (“breakthrough”) was reported to increase over time (22). Finally, long-term follow-up would increase the probability of a patient to develop cardiovascular comorbidity which is known to aggravate outcome in APS patients (7, 23, 24). Other plausible explanations for the relatively higher rate of thrombosis in our study may be attributed to the selection of patients only with pAPS, the high rate of triple aPL positivity (57%) documented in our cohort (8), and the as-yet unidentified genetic or environmental factors specific to Israeli patients.

Currently, there are gaps in our knowledge of APS patterns of recurrence. In this study, we attempted to identify features associated with recurrent thrombosis that may allow a more tailored approach to follow-up and treatment of pAPS patients. We identified 4 patterns of thrombotic recurrence, namely, “arterial”, “venous”, “combined”, and “breakthrough”, each associated with different risk factors that can be easily evaluated in any APS clinic explicitly APS-presenting symptom, patients’ age, aPL isotypes, and comorbidities (e.g., non-criteria manifestation, hypertension, and others).

In our cohort, we found that the significance clinical predictive factor for arterial, venous, or combined recurrence was the nature of the initial thrombosis: arterial recurrence correlating with arterial events at diagnosis and venous and combined recurrence correlating with venous thrombosis at presentation. Similar correlations between APS-defining events and type of recurrence have been previously reported in some cohorts (6, 21, 25, 26) but not in all studies (8, 22).

A prominent feature among APS patients with all types of recurrence was the presence of heart valve disease. While this manifestation is not considered an APS criterion, our cohort stresses the clinical importance of this non-criteria feature. Currently, little is known about the role of heart valve disease as a prognostic factor of APS. One report has stated that in obstetric APS patients heart valve disease was associated with an elevated risk of thrombosis (27). Whether heart valve disease in APS is a consequence of thrombotic complication or a manifestation of immune activation is yet to be determined; nonetheless, it seems to be a strong predictor of further event and thus it seems prudent to include regular assessment of the heart valves in primary APS patients.

Herein, another interesting link with pattern of APS recurrence was the presence of specific aPLs as well as the gravity of triple aPL positivity. The anti-phospholipid antibodies are not only nowadays regarded as classification criteria of APS but also clearly linked with the pathogenesis and severity of disease (8, 28, 29). In particular, Pengo *et al.* showed that the triple positivity of aPL is predictive of worst outcomes (8). Others demonstrated that IgG aCL is predictive of new thrombotic events (7) whereas LAC was related to adverse obstetric outcomes (30, 31). Recently, we reported a correlation between criteria and non-criteria aPL profiles and APS phenotype at presentation (29). In the current study, we found a small but significant association between high-titer aB2GPI of the IgM isotype and recurrence arterial thrombosis, while venous recurrence was associated with aB2GPI and aCL of the IgG isotypes as well as high titers. This is in agreement with other reports, especially of the association between aPL-IgM isotypes with older age, arterial events (8, 29), and stroke (32). Interestingly, elevated IgG subtypes (both at normal and very high titers) are associated.

The aGAPSS is nowadays the most validated score for APS recurrence and in our study was ubiquitous among all phenotypes. Previous studies have shown that the aGAPSS score is useful to predict the thrombosis (11) and rate of thrombosis among obstetric APS patients (27). The APS ACTION study had demonstrated this score to be significant in predicting thrombotic recurrence (13), although relatively lower scores for aGAPSS were reported in this study population for those with and without recurrence. This stands in agreement with our data, and it is our belief that the relatively higher aGAPSS scores in our cohort provide another explanation to the relatively high rate of thrombosis observed in this study.

Interpreting aGAPSS in regard to therapeutic decisions might be somewhat of a challenge, as this score is based on two pillars: one is the cardiovascular risk factors and the other is the serological positivity of aPL antibodies. Tailoring the therapy according to this score as well as patient phenotypes might be a feasible option in APS clinics. Nonetheless, it may be postulated that addressing comorbidities in the therapeutic arsenal for APS is of the essence, while other immunomodulatory interventions might be considered as treatments with hydroxychloroquine (33, 34) or anti-CD20 monoclonal antibodies (35) for patients at a higher risk of recurrence.

Last but not least, tailoring of APS therapy according to the initial thrombotic event had been proposed in the past especially in regard to arterial thrombosis (36). More recently, in close proximity to the end of our study in 2019, the new European League against Rheumatism (EULAR) recommendations for management of APS were published, in which intensifying therapy for patients with recurrent events and potentially for those presenting with arterial thrombosis and/or a high-risk aPL profile was also suggested (4). This stands in support with our data, as the vast majority of patients with the “breakthrough” pattern in this study were treated with anticoagulant ± antiplatelet therapies at the usual doses. Intensifying anti-thrombotic therapy is frequently limited by the fear of increased bleeding. In this regard, the overall rate of any bleeding in our cohort was 0.65% annually and the major bleeding rate was 0.97 events per 100 patient years. According to a recent published analysis, this rate of bleeding can be considered as low in comparison to the general population of patients treated with anticoagulants (37).

Our study has several limitations that derived from its retrospective nature. These include incomplete and possible inaccurate reporting especially given the prolonged duration of follow-up and the inability to verify patient adherence to antithrombotic treatments. In light of the retrospective design of the study, the definition of thrombosis despite preventive therapy was defined by the treating physician. INR levels were too often uncertain at the time of thrombosis; thus, we have chosen to rely on treating physician appraisal. Notably, the breakthrough pattern was not compared to patients with recurrence but not of the breakthrough pattern, as the latter was a too-small group and thus was compared to patients with no recurrence at all. However, we believe that these limitations are compensated for by the multicenter nature of this study which is relatively large and the well-defined cohort of primary APS patients. In addition, the similarities regarding mortality rates, thrombosis recurrence, and incidence of cAPS between our population and other published cohorts support the validity of our data.

In summary, and in agreement with previous studies (9, 13, 22, 38), we found that during a relatively long follow-up almost half (46%) of pAPS patients suffered a new thrombotic event. Given the challenge to foresee the risk of APS recurrence, we identified 4 phenotypes of recurrence: arterial, venous, combined, and breakthrough. Each of these patterns was linked with specific risk factors that can be assessed in every APS clinic and may enable physicians to better allocate patients at a higher risk and potentially tailor interventions accordingly.

## Data Availability Statement

The raw data supporting the conclusions of this article will be made available by the authors, without undue reservation.

## Ethics Statement

The studies involving human participants were reviewed and approved by the Sheba Medical Center Helsinki Committee. Written informed consent for participation was not required for this study in accordance with the national legislation and the institutional requirements.

## Author Contributions

SN and NA-L contributed to the conception and design of the study. SN, SH, and NA-L organized the database. SN performed the statistical analysis. SN and NA-L wrote the first draft of the manuscript. ME wrote sections of the manuscript. All authors contributed to the manuscript revision and read and approved the submitted version.

## Funding

Publication fees were funded by the grant from the Bayern Scholarship for research in the field of thrombosis and coagulation disorders granted to SN by Israeli Association of Internal Medicine.

## Conflict of Interest

The authors declare that the research was conducted in the absence of any commercial or financial relationships that could be construed as a potential conflict of interest.

## Publisher’s Note

All claims expressed in this article are solely those of the authors and do not necessarily represent those of their affiliated organizations, or those of the publisher, the editors and the reviewers. Any product that may be evaluated in this article, or claim that may be made by its manufacturer, is not guaranteed or endorsed by the publisher.
